# Seasonal variation in sleep time: jackdaws sleep when it is dark, but do they really need it?

**DOI:** 10.1007/s00360-023-01517-1

**Published:** 2023-10-03

**Authors:** Sjoerd J. van Hasselt, Massimiliano Coscia, Giancarlo Allocca, Alexei L. Vyssotski, Peter Meerlo

**Affiliations:** 1 Groningen Institute for Evolutionary Life Sciences, University of Groningen, Groningen, The Netherlands; 2 The Florey Institute of Neuroscience and Mental Health, Parkville, VIC Australia; 3 School of Biomedical Sciences, The University of Melbourne, Parkville, VIC Australia; 4 Somnivore Pty. Ltd., Bacchus Marsh, VIC, Australia; 5 Institute of Neuroinformatics, University of Zurich and Swiss Federal Institute of Technology (ETH), Zurich, Switzerland

**Keywords:** Sleep, Birds, Jackdaws, Seasonality, EEG

## Abstract

Sleep is an important behavioural and physiological state that is ubiquitous throughout the animal kingdom. Birds are an interesting group to study sleep since they share similar sleep features with mammals. Interestingly, sleep time in birds has been shown to vary greatly amongst seasons. To understand the mechanisms behind these variations in sleep time, we did an electro-encephalogram (EEG) study in eight European jackdaws (*Coloeus monedula*) in winter and summer under outdoor seminatural conditions. To assess whether the amount and pattern of sleep is determined by the outdoor seasonal state of the animals or directly determined by the indoor light–dark cycle, we individually housed them indoors where we manipulated the light–dark (LD) cycles to mimic long winter nights (8:16 LD) and short summer nights (16:8 LD) within both seasons. Jackdaws showed under seminatural outdoor conditions 5 h less sleep in summer compared to winter. During the indoor conditions, the birds rapidly adjusted their sleep time to the new LD cycle. Although they swiftly increased or decreased their sleep time, sleep intensity did not vary. The results indicate that the strong seasonal differences in sleep time are largely and directly driven by the available dark time, rather than an endogenous annual clock. Importantly, these findings confirm that sleep in birds is not a rigid phenomenon but highly sensitive to environmental factors.

## Introduction

Sleep and sleep behaviour are ubiquitous throughout the animal kingdom, ranging from invertebrates like jellyfish and insects (Siegel 2008; Lesku et al. 2009) to vertebrates like mammals and birds (Borbély and Neuhaus 1979; Martinez-Gonzalez et al. 2008; Coolen et al. 2012; Van Hasselt et al. 2020a, b). The wide-spread occurrence of sleep at various levels of evolution across the animal kingdom indicates that sleep must serve an important function. Moreover, it is thought that sleep is homeostatically regulated indicating that a loss of sleep is recovered in a dose-dependent manner (Borbély and Achermann 1999; Benington 2000; Deboer 2013).

Birds are an interesting group of species to study sleep because they share the two sleep states with mammals: rapid-eye-movement (REM) sleep and non-REM (NREM) sleep (Borbély and Neuhaus 1979; Szymczak 1985; Beckers and Rattenborg 2015). Moreover, the functional mechanisms of NREM and REM sleep show in some ways similarities between mammals and birds (Lesku et al. 2011; Scriba et al. 2013; Rattenborg and Martinez-Gonzalez 2015).

Whilst studies in particularly mammals under laboratory condition suggest that sleep is a tightly regulated state (Deboer 2013), recent studies in birds show large variation in qualitative and quantitative expression of sleep under (semi-)natural conditions and at different phases of the year. The seasonal changes in sleep in birds, most of which are day-active, often consist of more sleep during the winter, when the nights are longer, compared to summer, when the nights are shorter (Szymczak 1986b; Steinmeyer et al. 2010; van Hasselt et al. 2020a, b). The largest variation in sleep time between winter and summer was measured in the European starling (5 h more sleep in winter compared to summer) (van Hasselt et al. 2020a, b). Besides different amounts of sleep across seasons, the robustness of circadian rhythmicity is also season-dependent, for instance in the arctic herbivorous barnacle goose (Van Hasselt et al. 2021a, b; van Hasselt et al. 2022). In extreme cases, birds can entirely forgo with sleep during the breeding season such as the pectoral sandpiper that displays nearly non-stop waking and activity for about two weeks (Lesku et al. 1979).

The mechanisms underlying these seasonal differences in sleep time and distribution in birds are poorly understood. It has been shown that some animals have endogenous timing systems or clocks that can generate circannual rhythms in physiology and behaviour, for instance migration in birds (Gwinner 1996) and hibernation in small mammals (Pengelley et al. 1978). However, it remains unclear whether the changes in sleep time and sleep distribution across seasons in birds are driven by such a circannual clock system or whether they are a direct consequence of environmental factors, i.e. a sleep-suppressing effect of light.

In the current study, we examined seasonal variation in sleep in the European jackdaw (*Coloeus monedula*). We measured sleep in jackdaws under seminatural outdoor conditions in winter and summer to assess seasonal variation in sleep time and sleep architecture. We then moved summer and winter birds indoors under controlled opposite photoperiods to assess if the amount and pattern of sleep is determined by the outdoor seasonal state of the animals or directly determined by the indoor light–dark cycle.

## Methods

### Animals and housing

Eight European jackdaws (4 males; 4 females) were used for this study. The animals were retrieved from nest boxes in a wild jackdaw colony as nestlings (30 days old). They were transferred to seminatural outdoor enclosures (length = 500 cm, width = 400 cm, height = 230 cm) and separated by sex (2 enclosures, one with 4 males and one with 4 females). The animals were hand-fed by the experimenter 7 times a day using specific bird food (Versele-laga, NutriBird A21, Deinze, Belgium) between natural sunrise and sunset up to an age of 45 days. During this time, the birds were slowly accustomed to the water pond and food tray present in the enclosure. Food (Kasper Faunafood item number 6659; Woerden, The Netherlands) and water were present ad libitum.

### Surgery

A minimum of two weeks before the experiment, the animals underwent surgery for implantation of epidural electroencephalogram (EEG) electrodes. The birds were given isoflurane (1.5–2%) as anaesthetics and meloxicam (0.022 ml; 0.5 mg/kg) as analgesic. Whenever the bird was stable under anaesthesia and did not respond to pain stimulus, we started the surgery. After carefully exposing the crania, four holes (0.5 mm in diameter) were drilled to the level of the dura mater. Two frontal gold-plated electrodes (BKL Electroninc 10120538, Lüdenscheid, Germany) were inserted to the level of the dura mater 2 mm lateral of the midline covering the hyperpallium. Another two gold-plated electrodes were placed caudally over the cerebellum 2 mm lateral of the midline that served as a ground and reference. In addition, a flexible wire was inserted on to the nuchal muscle for recording an electromyogram (EMG). Finally, a screw (1 mm in diameter) was inserted over the right hemisphere 4 mm rostral of the frontal left electrode that served as an anchor point for the implant. The electrodes were soldered to a 7-channel connector (BKL Electroninc 10120302, Lüdenscheid, Germany) and all the electrodes were fixated on the skull using dental cement (Heraeus Kulzer, Hanau, Germany). A light-weight protective plug was attached to the connector of the implant to protect the pins (BKL Electroninc 10120602, Lüdenscheid, Germany). After the surgery, the animals were brought back to the outdoor enclosure for recovery.

### Sleep recordings

To record and store EEG data, a data logger was attached to the implant (Neurologger 2A; Evolocus, Tarrytown, NY, USA). The logger contained an on-board memory chip (2 GB) and a 3-axis accelerometer (LIS302DLH; STMicro-electronics Geneva, Switzerland) Using the miniature data logger, we recorded and stored EEG, EMG and accelerometry data with a sampling frequency of 100 Hz. The logger was powered by two ZA13 1.45 V batteries (Ansmann ZA13; Assamstadt, Germany) that enabled the logger to run for approximately 4.5 days.

**Fig. 1:**
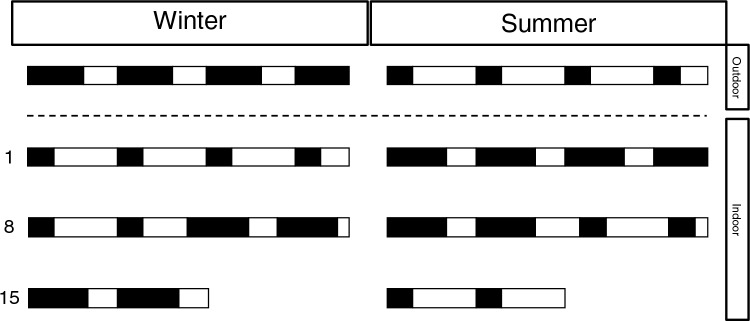
Schematic overview of the photoperiod manipulation in winter (left) and summer (right) where the black bars denote night and the white bars denote day. The animals were group-housed and exposed to natural light–dark cycles during the outdoor recordings. During the indoor recordings, the animals were housed individually. The numbers on the left indicate the day relative to the start of the indoor housing. In between every row of the indoor recordings is 7 days

After a minimum of two weeks of recovery from surgery, sleep was recorded in the outdoor aviaries at the beginning of February. During these recordings, the birds were group-housed and separated by sex over 2 outdoor aviaries. The loggers were attached to the implants before noon to ensure undisturbed sleep during the first night. After the 3-day outdoor recordings, the animals were directly transferred indoors and housed individually in wooden boxes (length = 79 cm, width = 60 cm, height = 60 cm) where we did a series of photoperiod switches to understand the seasonal regulation of sleep. At the start of the indoor recordings, we set the photoperiod opposite to that of the outdoor season (18:6 LD cycle to mimic a summer photoperiod). After 4 days of the indoor recording, the loggers were removed and the animals remained in their indoor enclosures to further habituate to the summer photoperiod. After one week, sleep was measured again for 4 days to see whether there would be gradual changes in sleep in response to the new LD cycle. After two days of that recording, the photoperiod was shifted back to an LD cycle that matched the outdoor situation (6:18 LD cycle) for the remaining 2 days. After one week of habituation to the last shift, we measured sleep again for a total of 3 consecutive days. After that recording, the animals were released back in their outside enclosures. At the beginning of June, we exposed the birds to the same number of LD shifts but in the opposite direction (Fig. 1). Using this series of shifts, we could compare sleep between and within each season under habituated artificial LD cycles of both long and short nights. Measuring sleep after each shift helped us understand how quickly the animals adapt to the new LD cycles. Throughout the indoor recordings, the ambient temperature was constant at 21 °C. The average daily outdoor temperature for the winter recordings was 6.3 ± 0.66 °C and for the summer recordings was 14.9 ± 0.6 °C.

### Data analyses

After the experiments, the data were downloaded from the loggers and were further processed and analysed. Sleep was scored on a 4-s basis using an automated sleep scoring programme (Somnivore Pty Ltd., Bacchus Marsh, VIC, Australia). The programme used all available electrophysiological channels (EEG + EMG + accelerometry) to determine the vigilance states based on a manually scored subset of 4-s epochs (± 100 epochs per state). The manual training was done for every individual EEG recording. A state was scored when the majority of the signals exhibited that state for the majority of the epoch. Wake was scored whenever the recording showed high frequency, low amplitude EEG activity, often in combination with head movements visible in the accelerometer channels. NREM sleep was scored when the recording showed low frequency, high amplitude EEG activity, at least twice as high as the EEG amplitude during wakefulness, together with a lack of head movements in the accelerometer channels. REM sleep was scored when the recording showed low amplitude, high frequency EEG activity without noticeable head movements in the accelerometer or sometimes with signs of head drops indicative of reduced muscle tone. Based on the automatic scorings, we calculated the amounts of NREM and REM sleep per hour. The programme has been validated for EEG recordings in several bird species, including pigeons (Allocca et al. 2019), barnacle geese (Van Hasselt et al. 2021a, b) and Australian magpies (Connelly et al. 2020). We compared five manually scored 24-h baseline recordings with the autoscore and found an overall scoring agreement of 93.8 ± 0.4%. Furthermore, we calculated *F*-measures for every vigilance state between the manual and autoscore that reflects the precision, sensitivity and specificity for each state (Allocca et al. 2019). These values were 0.98 ± 0.02 for Wake, 0.95 ± 0.02 for NREM sleep and 0.89 ± 0.03 for REM sleep.

To obtain clean EEG signals, a second round of scoring was performed on the data that did not affect sleep staging but might affect the spectral quality of the sleep stages. All epochs that showed movement artefacts that resulted in peaks that were twice as high as their respectively clean vigilant stage were labelled as artefacts. All artefact-free NREM sleep epochs were then subjected to a fast Fourier transformation (FFT). The FFT analysis resulted in 256 frequency bins with a bin width of ~ 0.2 Hz. All individuals that had successful recordings in the outdoor group-housed conditions were included in the FFT analysis (winter: *n* = 6; summer: *n* = 7). For every individual, all NREM sleep FFT values were normalised to the average night-time outdoor baseline values for every frequency bin. A broad range of frequency bins (1.5–25 Hz) were then averaged that have been suggested to reflect sleep pressure in birds (Martinez-Gonzalez et al. 2008; Van Hasselt et al. 2020a, b). Furthermore, these power values were subsequently averaged per hour in parallel to the number of NREM sleep epochs. Next to NREM sleep EEG power, we calculated the cumulative NREM sleep EEG energy by calculating the product of NREM sleep EEG power and number of NREM sleep epochs. All cumulative NREM sleep EEG energy values were normalised to the total NREM sleep EEG energy of the outdoor recordings.

### Statistics

All data were analysed in the statistical programming language R, and linear mixed effect models were made using animal ID as a random effect (R Development Core Team 3.0.1. 2013; Bates et al. 2015). Whenever the random component did not contribute to a better model using the likelihood ratio test, a more simplistic linear regression model was used. Whenever a model was significant using an ANOVA test, we computed a post hoc analysis using the lsmeans package (Lenth 2016). All data in text and figures are presented as mean ± standard error of the mean (SEM).

## Results

**Fig. 2:**
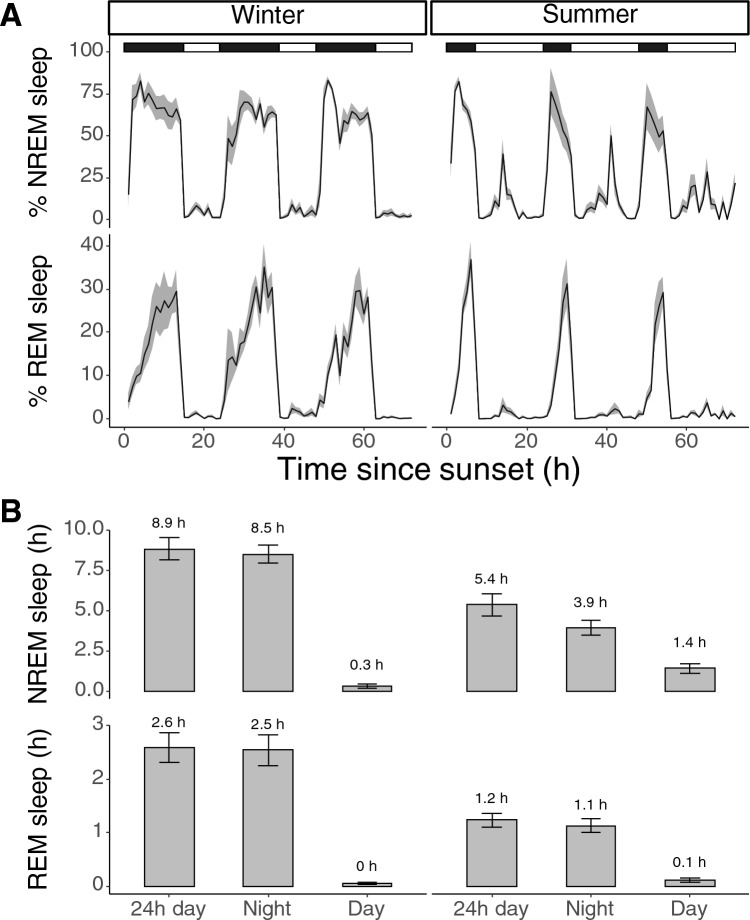
Seasonal differences in sleep architecture between natural winter and summer conditions. Most sleep occurred during the night time (black and white bar on top denotes the night and day respectively). NREM sleep time was greatly reduced from winter to summer by 3.8 ± 1.1 h. REM sleep time was reduced by 1.5 ± 0.3 h. The reduction in night length between winter and summer resulted in more daytime naps of NREM sleep

The Jackdaws showed clear differences in the amount of NREM and REM sleep between winter and summer under semi-natural outdoor conditions (Fig. 2). During a 24-h day in winter (February), the jackdaws spent  8.9 ± 0.7 h in NREM sleep and 2.6 ± 0.3 h in REM sleep in the outdoor enclosure. During a 24-h day in summer (June), the birds spent 5.4 ± 0.7 h in NREM sleep and 1.2 ± 0.1 h in REM sleep. This resulted in 3.8 ± 1.1 h more NREM sleep and 1.5 ± 0.3 h more REM sleep in winter compared to summer. The proportion of REM sleep relative to total sleep time was 23.3 ± 3.1% in winter and 19.4 ± 1.1% in summer (*p* = 0.3, lmer model). Whilst the overall amount of sleep per 24-h cycle was reduced in summer compared to winter, the amount of day time sleep was larger in summer, i.e. 0.4 ± 0.1 h in winter and 1.6 ± 0.3 h in summer (Fig. 2B). Moreover, there were no significant differences in sleep time between males and females (*p* = 0.9, lm model).

**Fig. 3:**
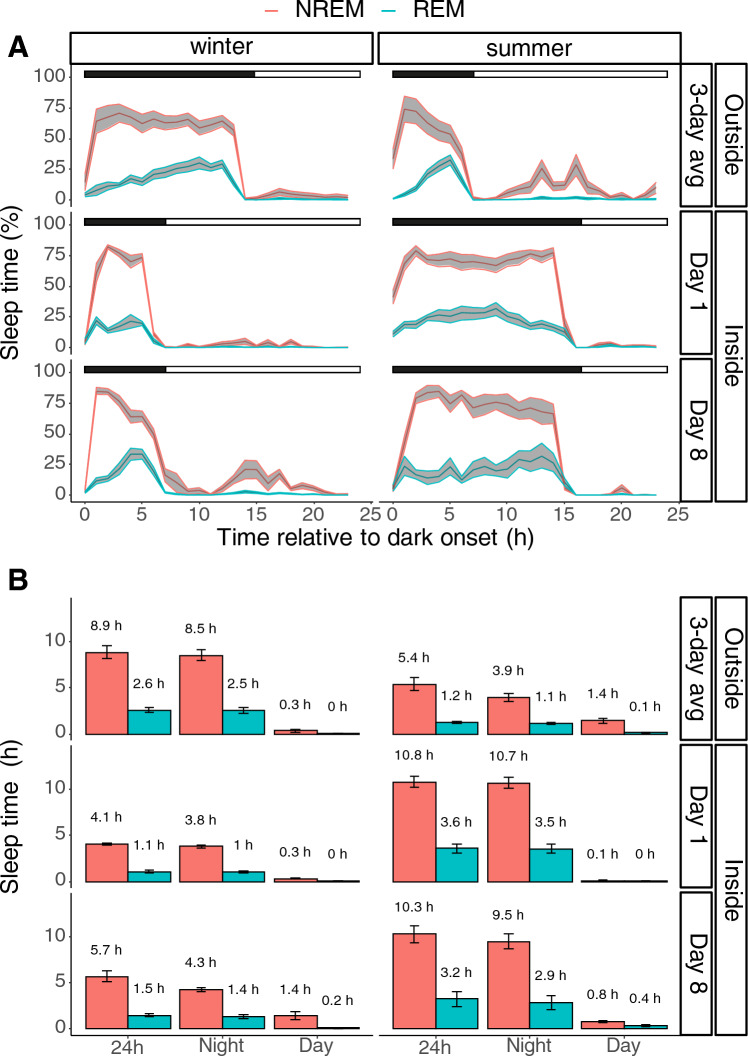
Daily distributions of NREM sleep (red) and REM sleep (blue) during a winter physiological state (left) and summer state (right). The bars on top represent the light–dark cycle (black denotes the night and white denotes the day). The birds were transferred from the natural group-housed outdoor enclosures to individual indoor cages where they were exposed to a photoperiod that mimicked the opposite season. Sleep was measured during the first day of transition and during the 8th day of acclimatisation to the new photoperiod (Day 1, Day 8, respectively). The birds showed a direct effect of the new light–dark cycle. There were no significant differences in sleep distribution over the 24-h day between Day 1 and Day 8 independent of physiological state (winter: *p* = 0.14; summer: *p* = 0.5, posthoc test after lm model; **A**). However, for birds in their internal physiological winter state the total amount of 24-h sleep at Day 8 was 1.6 h more than at Day 1 (*p* < 0.001 posthoc test after lmer model; **B**). Birds in their internal physiological summer state did not show significant differences in sleep time between day 1 and day 8. During the winter state, birds had a total sleep time that was 4.6 ± 0.5 h less in the indoor summer conditions compared to the outdoor conditions (*p* < 0.001; posthoc test after lmer model). During the summer state, birds had a total sleep time that was 7.1 ± 0.8 h more in the indoor winter conditions compared to the outdoor conditions (*p* < 0.001; posthoc test after lmer model)

When the animals were transferred to their indoor enclosures, they showed a fast and immediate response to the altered light–dark condition (Fig. 3, outside vs indoor day 1). Winter birds from an outdoor condition with short days and long nights that were moved to an indoor condition with long days and short nights immediately adapted a summer sleep pattern. Vice versa, summer birds from an outdoor condition with long days and short nights that were moved to an indoor condition with short days and long nights instantaneously reversed to a winter sleep pattern. There were no differences in sleep distribution over the 24-h day between direct exposure to the new photoperiod compared to the sleep patterns after 8 days of habituation (winter: *p* = 0.14; summer: *p* = 0.5, post hoc test after lmer model; Fig. 3A, indoor day 1 vs day 8). However, birds in their internal physiological winter state under a summer photoperiod showed 1.6 h more NREM sleep on day 8 compared to day 1 (5.7 h vs 4.1 h, respectively; *p* < 0.001 post hoc test after lmer model). Birds in their internal physiological summer state under a winter photoperiod showed no significant differences in sleep time between day 1 and day 8. Overall, sleep remained largely confined to the available dark period under all conditions. This resulted to that birds had a 24-h total sleep time that was 4.6 ± 0.5 h less in the indoor summer conditions compared to the outdoor winter conditions (*p* < 0.001; post hoc test after lmer model). Oppositely, birds had a 24-h total sleep time that was 7.1 ± 0.8 h more in the indoor winter conditions compared to the outdoor summer conditions (*p* < 0.001; post hoc test after lmer model).

**Fig. 4:**
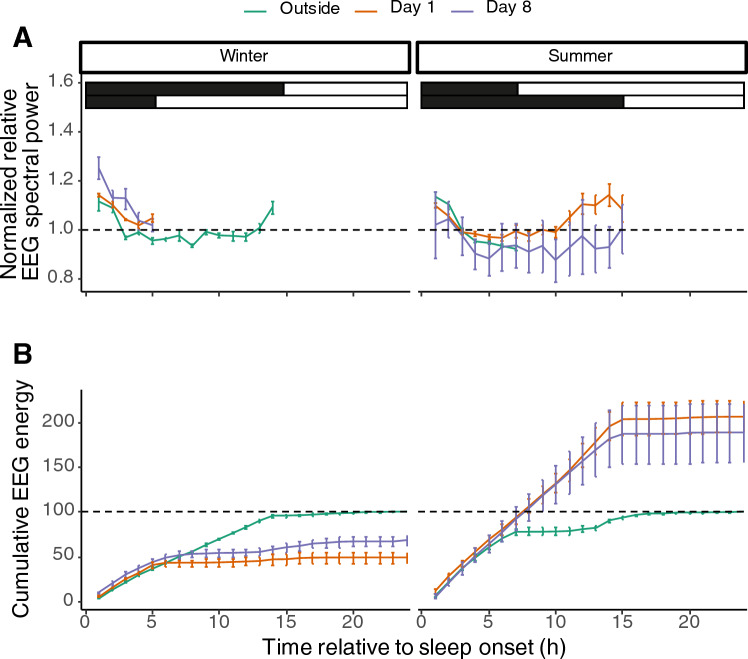
Changes in a broad range of spectral power (0–25 Hz) in NREM sleep relative to the outdoor night between birds in the winter state (left) and summer state (right). The bars on top represent the light–dark cycle (black denotes the night and white denotes the day) where the upper bars denote the outdoor natural LD cycle and the lower bars denotes the artificial indoor LD cycle. The animals showed an increase of spectral power at the start of the night that dissipates over the course of the night. There were no differences between NREM sleep spectral power between the three days in both seasons (**A**). The cumulative NREM sleep energy (**B**), that is the product of NREM sleep time and NREM sleep EEG spectral power, shows that birds in their internal physiological winter state partially compensated to the sleep loss due to the short summer nights by sleeping deeper and more (*p* < 0.005; lmer model; panel B left). Birds in their internal physiological summer state showed a significant increase in NREM sleep energy due to an increase in additional NREM sleep time (*p* < 0.001; lmer model; **B** right)

The next step was to see whether NREM sleep spectral power was altered across the seasons and under different photoperiods. For both seasons, we normalised the NREM sleep spectral power to the average outdoor night time spectral power of NREM sleep. For all conditions, the animals showed  a peak in spectral power at the start of the night that slowly  decreased over the course of the dark phase. Birds from an outdoor winter condition that were moved to a short summer night indoors did not show a significant difference in the pattern of spectral power across the night (Fig. 4A left panel). Likewise, birds that were moved from a condition of short summer nights outside to long winter nights inside did not show changes in NREM sleep spectral power (Fig. 4A right panel).

We then calculated the cumulative sum of NREM sleep EEG spectral energy, which is the product of NREM sleep time and NREM sleep spectral power. Birds that came from long outdoor winter nights and were moved to a short indoor summer night did not reach the same daily cumulative NREM sleep energy, neither on the first day, nor on the 8^th^ day indoors (Fig. 4B, left panel). On day 8, there is a significant higher NREM sleep energy at the end of the day (*p* < 0.005, lmer model), but this does not compensate for the induced sleep loss by the indoor LD with short nights compared to the natural outdoor winter condition (*p* < 0.001, lmer model). Birds in their internal physiological summer state that moved to the indoor LD with long nights had an immediate increase in NREM sleep energy at the end of the 24-h day on day 1 and day 8 compared to the outdoor recordings (*p* < 0.001, lmer model; Fig. 4B, right panel).

**Fig. 5:**
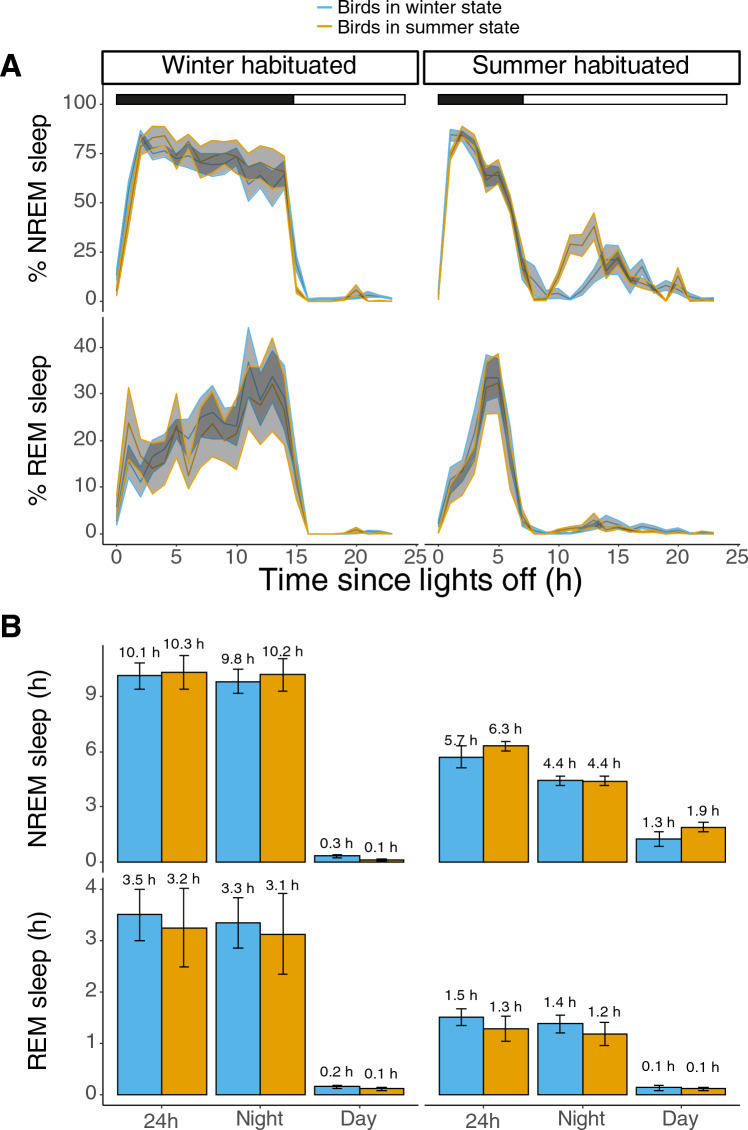
Sleep architecture of birds that are housed indoors that were habituated under a winter photoperiod (left panel) and summer photoperiod (right panel) in an internal physiological winter state (blue) or internal physiological summer state (orange). The bars on top represent the light–dark cycle (black denotes the night and white denotes the day). There were no significant differences in the hourly values of both sleep states between birds that were in an internal physiological winter or summer state

After 8 days under an indoor LD cycle that was opposite to their original seasonal outside condition, we changed the indoor LD cycle to match the original outdoor season. We then compared sleep patterns under indoor LD with short nights for summer and winter birds, and we compared indoor LD with long nights for summer and winter birds. With this, we assessed whether habituated indoor sleep pattern would be affected by the original outdoor season. Within both LD cycles with either long or short nights, there were no differences in the distribution of NREM and REM sleep between birds in their internal physiological winter or summer state (Winter habituated: *p* = 0.98; Summer habituated: *p* = 0.82; lmer model; Fig. 5A).

## Discussion

This study shows that Jackdaws under seminatural conditions display strong differences in the amount and pattern of sleep between winter and summer. Both in winter and summer, the birds spent most of the night time asleep and the day time awake. This translated to a 4.9 h lower amount of sleep in June with short summer nights compared to February with long winter nights. Transferring the birds to controlled indoor conditions with different LD cycles suggests that the seasonal differences in sleep are largely driven by the cycle of light and darkness, i.e. the length of the night, and not by an internal seasonal programme.

During the undisturbed outdoor conditions, the jackdaws displayed a large amount of REM sleep of 21.4% per TST. These values of REM sleep are larger than earlier reported sleep data on the jackdaw (2.4% of TST) (Szymczak 1986a). This discrepancy might be explained by the use of different recording techniques. However, the rook and Australian magpies also show lower levels of REM sleep 1.8 and 8.2% per TST, respectively (Szymczak 1987; Johnsson et al. 2022). Higher levels of REM sleep are not uncommon for birds as shown by zebra finches and budgerigars that express 23% and 26.5% of REM sleep per TST, respectively (Low et al. 2008; Canavan and Margoliash 2020). It is still a topic of debate what causes the variation in REM sleep amongst birds.

Whilst in our jackdaws, the amount and distribution of sleep was largely determined by the available dark time, we did find a slight redistribution of sleep across the seasons. Particularly, we found that both short summer nights outdoors and short dark phases indoors were associated with in an increase in daytime naps. This might suggest that in midsummer when nights are the shortest, the available dark time is not fully sufficient for the sleep they need. This finding is in agreement with our earlier studies in starlings, which also displayed an increase in mid-day sleep during summer (van Hasselt et al. 2020a, b).

The seasonal difference in amount of sleep per 24-h cycle we observed in the jackdaws is in agreement with other studies in diurnal songbirds. For example, rooks, another crow species related to the jackdaws, showed under semi-natural conditions a reduction of 3.4 h in sleep time in summer compared to winter (Szymczak 1987). Also, the European starling under seminatural conditions showed 5 h less NREM sleep per day in summer compared to winter (van Hasselt et al. 2020a, b) and the duration of the night was as a strong predictor for sleep time (Szymczak 1986b; van Hasselt et al. 2020a, b). Studies in wild blue tits and great tits reported 5 h more resting behaviour per day in winter compared to summer (Steinmeyer et al. 2010; Stuber et al. 2015). Whilst the latter studies in tits relied on video recordings to assess rest and did not assess EEG-based sleep, the results point in the same general direction as our current study.

In mammals, on the other hand, data on changes in sleep across seasons and different photoperiods are scarce and do not paint a simple picture. Actigraphy studies on humans from natural pre-industrial settlements showed a reduction in resting time of 2 h in summer compared to winter (Samson et al. 2017) and even humans in post-industrial societies that experience artificial lights year-round have 25 min less rest in spring compared to winter (Mattingly et al. 2021). Also, an EEG-based experimental study in human subjects under laboratory conditions showed that extending the dark phase from 8 h per day to 14 h per day to mimic long winter nights not only induced a bimodal sleep pattern but also increased the overall sleep time with slightly more than an hour (Wehr 1992). Whilst these actigraphy studies in humans suggest seasonal changes in sleep that are in line with the findings in birds described above, studies in other mammals do not show clear changes in sleep time with season or photoperiod. For example, the diurnal rodent *Eutamias sibiricus* adapted to LD cycles with different photoperiods ranging from LD 18:6 to LD 6:18 showed redistribution of sleep and wakefulness over light and darkness but overall sleep time was not significantly affected (Dijk and Daan 1989). Similarly, nocturnal Djungarian hamsters adapted to either LD 18:6 or LD 6:18 showed differences in the distribution of sleep across the day but the total amount of sleep was unchanged (Deboer and Tobler 1996; Palchykova et al. 2003). The latter findings may suggest that under different environmental conditions and in different seasons, these species defend their total sleep time.

To assess whether the seasonal variation in sleep in jackdaws is regulated by an endogenous seasonal clock or perhaps is a direct effect of environmental factors, e.g. a sleep-inhibiting effect of light, we exposed the birds in winter and summer to artificial LD cycles that were the reverse of the natural cycle. When sleep time is regulated by an endogenous circannual clock, we expected the amount of sleep to be unaffected by the transfer to the artificial LD cycle. Oppositely, when sleep is not driven by a circannual clock but directly driven by environmental factors, i.e. light and darkness, we expected that moving the animals to the artificial LD would immediately change their sleep patterns and sleep time. We found that the latter was true. Moving the jackdaws indoors, and housing them under an LD that was opposite of the natural cycle outdoors, resulted in an immediate change in their sleep pattern and sleep time. In each indoor condition, sleep was largely confined to the dark hours and thus total sleep time was primarily driven by the length of the dark phase, not by the natural outdoor season. The sleep-suppressing effect of longer exposures to light are in agreement with other studies that found that birds are particular sensitive to artificial light at night (Aulsebrook et al. 2018, 2020a, b; van Hasselt et al. 2021a, b).

One might argue that birds could still have an endogenous programme regulating sleep time across the year that is temporarily masked by direct sleep-inhibiting effects of light. However, this is not supported by the fact that the altered sleep time under the artificial indoor LD cycle was stable over a period of 8 days. If an endogenous drive for long sleep in winter birds would be masked by an acute sleep-inhibiting effect of artificial light, then one could expect that over a period of a week indoors the endogenous drive would push animals back to getting more of the long winter sleep they presumably need. Whilst indeed after 8 days under the indoor short night condition the birds got 1.6 h more sleep than on the first day under short night, the sleep time on day 8 was still far less than in the outdoor condition. Also, if short sleep in summer birds would be endogenously regulated and adapted to their needs, then there would be no reason for an acute and large increase in sleep when housed indoors under artificial long nights. Altogether, the data provide no evidence for an endogenous programme or clock regulating the large seasonal variation in sleep time. Instead, the results of our study suggest that jackdaws simply sleep when it is dark.

The seasonal variation in sleep time in some species but not in others is an interesting finding in the context of sleep homeostasis and sleep function. The fact that some species, particularly small mammals, such as hamsters and squirrels, show changes in sleep patterns under different photoperiods but maintain total sleep time (Dijk and Daan 1989; Deboer and Tobler 1996), could suggest that there is a homeostatic need or drive for a fixed amount of sleep that is defended in these animals, independent of the seasonal environment. The fact that in various other species sleep time changes across the year, could be explained by a change in sleep need in these species, which might be related to seasonal changes in behaviour, metabolism and brain function. Based on the common view that sleep is a process that supports recovery of the brain from wakefulness, it might be that the various species sleeping less in a summer state as mentioned above build up less need for recovery during their waking hours. Alternatively, it might be that some species in winter sleep more than they need reminiscent to the early concept of core sleep and optional sleep proposed by James Horne (Horne 1989). Perhaps jackdaws need a certain amount of essential core sleep that is fairly similar in winter and summer, but in winter they get many hours of optional sleep because there is little else to do during the long dark nights. In that case the amount of sleep does not reflect an increased need for sleep but rather a decreased need for wakefulness.

In summary, our data show that jackdaws under seminatural conditions display striking variation in sleep time across the year, with almost 5 h more sleep in winter than in summer. Transferring the animals indoors and exposing them to different LD cycles indicate that the strong seasonal differences in sleep time are largely and directly driven by the available dark time, rather than an endogenous annual clock. Importantly, these findings confirm that sleep in birds is not a rigid phenomenon but highly sensitive to environmental factors.

## Data Availability

Data are available upon request.
